# Safety and Preliminary Clinical Outcomes of Integrating Engineered Synthetic Lipid Vesicles (Biosomes) in a Skin Booster Protocol: A Prospective Observational Study

**DOI:** 10.3390/biomedicines14102172

**Published:** 2026-09-25

**Authors:** Antonella Franceschelli, Alessandra Procaccini, Deborah Subissati, Michela Pallini, Rob Knight, Maria Luisa Garo

**Affiliations:** 1 Master of Aesthetic Medicine, Unicamillus International Medical University, 00131 Rome, Italy; 2 Independent Researcher, Studio Medico Franceschelli, 00195 Rome, Italy; 3 WiQo S.p.A., 34015 Trieste, Italy; 4 Cellese Inc., Irvine, CA 92614, USA; 5 Biostatistics and Research Methodology Unit, Mathsly Research, 89900 Vibo Valentia, Italy

**Keywords:** skin booster protocol, engineered synthetic lipid vesicles, skin quality, aesthetic medicine

## Abstract

**Background**: Engineered synthetic lipid vesicles with biomimetic architecture have emerged as promising adjunctive agents in aesthetic medicine. Nevertheless, objective evidence evaluating their integration within standardized, minimally invasive skin booster protocols remains limited. The study aimed to assess the safety, tolerability, and preliminary clinical outcomes of incorporating engineered synthetic lipid vesicles (Biosomes) into a facial skin booster protocol. **Methods**: This prospective, single-center observational study included ten adult women aged 18–65 years. Participants received three treatment sessions at 21-day intervals, consisting of PRX PLUS application, microneedling and subsequent topical administration of engineered synthetic lipid vesicles (AnteAGE® Biosome Solution). Clinical assessments were conducted at baseline and 15 days after the final treatment session. Objective skin quality parameters were evaluated using the VISIA^®^ and Visioscope systems. **Results**: The combined protocol demonstrated a favorable tolerability profile, with minimal pain, discomfort, and burning sensation. Significant improvements were observed in texture-related parameters, pigmentation features, and redness values. Texture feature counts and scores were reduced by 20.0% and 25.8%, respectively (*p* ≤ 0.009). Redness features and scores decreased by 42.3% and 33.3% (*p* ≤ 0.022). Pigmentation-related parameters also showed significant reductions, while wrinkle and UV-related outcomes exhibited limited or non-significant changes. **Conclusions**: The integration of engineered synthetic lipid vesicles into a topical skin booster protocol demonstrated favorable immediate tolerability and was associated with significant improvements in selected objective skin quality parameters. Although preliminary, these findings suggest the potential value of this approach and support the need for further controlled studies to confirm its efficacy and durability.

## 1. Introduction

Skin aging is a multifactorial process resulting from the interplay between intrinsic factors, such as chronological aging and genetic predisposition, and extrinsic influences including ultraviolet radiation, environmental pollution, and lifestyle-related stressors [1]. These processes lead to progressive alterations in skin structure and function, primarily involving the dermal extracellular matrix and the complex signaling network between the epidermis and dermis. Clinically, these changes manifest as deterioration in skin texture, pigmentation homogeneity, vascular reactivity, sebaceous balance, and overall skin quality [2]. As a result, there is growing clinical demand for non-invasive or minimally invasive interventions capable of improving visible skin-quality parameters while preserving epidermal integrity and minimizing downtime. In this context, topical skin booster treatments have gained increasing relevance as pragmatic options in routine aesthetic practice, aiming to enhance skin quality through controlled cutaneous stimulation. These approaches are designed to activate dermal remodeling and epidermal–dermal communication while maintaining an acceptable tolerability profile and limited recovery time. In addition to their effects on dermal remodeling, such approaches may also transiently increase epidermal receptivity and intercellular signaling dynamics, creating a biologically favorable context for adjunctive therapies.

In parallel, recent years have seen growing interest in extracellular vesicles (EVs), particularly small extracellular vesicles (sEVs), as biologically active, cell-free agents in dermatology and aesthetic medicine. sEVs, a subpopulation of which are referred to as exosomes, are nanosized vesicles released by most eukaryotic cells and play a key role in intercellular communication through the transfer of proteins, lipids, and nucleic acids [3,4]. Along with naturally derived EVs, engineered synthetic lipid vesicles with EV-inspired architecture have emerged as an alternative approach, designed to replicate key structural and functional characteristics of EVs while enabling greater control over composition, scalability, and reproducibility. Experimental and early translational evidence suggests that EVs may modulate several processes relevant to skin homeostasis and regeneration, including inflammatory pathways, fibroblast activity, extracellular matrix remodeling, and epidermal–dermal signaling [5,6]. These mechanisms are similarly hypothesized to underpin the activity of engineered synthetic lipid vesicle systems with EV-inspired architecture, although clinical data in this area are currently limited.

From a clinical standpoint, EV-based formulations are currently explored predominantly as adjunctive therapies rather than standalone interventions. Their combination with established aesthetic procedures has been proposed as a strategy to enhance skin-quality outcomes, including improvements in texture, pigmentation uniformity, erythema, and overall skin appearance [7,8,9]. In addition to potential efficacy benefits, such adjunctive approaches may also influence early post-procedural responses, including inflammation, erythema, and patient-perceived tolerability. However, despite their increasing adoption in clinical practice, objective and methodologically robust data supporting their efficacy and tolerability within combination protocols remain limited.

The integration of topical facial skin boosters with EV-based approaches represents a biologically and clinically plausible strategy. Controlled epidermal activation induced by skin booster treatments may enhance tissue permeability and cellular responsiveness, potentially facilitating vesicle-mediated signaling and downstream regenerative effects, while preserving barrier function and minimizing procedural aggressiveness. Within this framework, vesicle-based technologies may act not only as regenerative signaling agents but also as modulators of the post-procedural microenvironment, influencing both early inflammatory responses and subsequent skin quality outcomes. Nevertheless, objective clinical evidence evaluating such combined protocols remains scarce, particularly regarding standardized instrumental assessments of skin quality and systematic evaluation of tolerability outcomes. The aim of the present study is therefore to investigate the safety, tolerability, and preliminary clinical effects of integrating engineered synthetic lipid vesicles (Biosomes) into a skin booster treatment protocol, with a specific focus on objectively measurable skin-quality parameters.

## 2. Materials and Methods

### 2.1. Study Design

This study was a prospective, single-arm pilot observational study conducted in a private outpatient aesthetic medicine setting between September and December 2025. The primary objective was to explore the clinical feasibility, safety, and preliminary effects of integrating an engineered synthetic lipid vesicle formulation (AnteAGE^®^ Biosome Solution, Cellese Inc., Irvine, CA, USA) with a facial skin booster protocol (PRX PLUS, WiQo S.p.A, Milan, Italy) under real-world clinical practice conditions. Given the exploratory nature of the study and the routine clinical use of the individual components, no control or comparator arm was included. PRX PLUS is a patented topical skin booster formulated to promote controlled cutaneous activation without inducing visible exfoliation or epidermal injury. In Italy, it is classified as a cosmetic product, and all procedures were therefore performed within the scope of standard aesthetic practice. In accordance with current national regulations, formal approval from an ethics committee was not required. All participants provided written informed consent prior to treatment, authorizing both the procedure and the anonymized use of clinical data for research purposes. The study was conducted in compliance with the principles of the Declaration of Helsinki and adhered to the STROBE guidelines for observational studies.

### 2.2. Participants

Participants were consecutively recruited into a prospective observational cohort at the outpatient practice. Eligible subjects were adult women aged 18 to 65 years presenting with clinical signs of facial photoaging and representing a range of Fitzpatrick skin phototypes. Inclusion criteria required willingness to complete the full treatment protocol consisting of three sessions and availability for both baseline and post-treatment evaluations according to the predefined schedule.

The enrollment of female participants only was intended to improve cohort homogeneity, considering known sex-related differences in skin physiology, including sebum production, wrinkle development, and pigmentation patterns. Exclusion criteria included: (1) pregnancy or breastfeeding; (2) recent facial aesthetic or dermatologic procedures within the preceding months; (3) active dermatologic conditions or infections involving the face; and (4) any known contraindication to microneedling or topical treatments. Eligibility was verified prior to initiation of the protocol to ensure prospective data collection.

### 2.3. Treatment Protocol

Each participant underwent three treatment sessions, scheduled at 21-day intervals, in line with routine clinical practice. At each session, PRX PLUS was applied to the entire face using a standardized approach. Two to three application passes were performed, with approximately 1 mL of product distributed across the treated area and gently massaged using a multilayering technique until full absorption. Upon completion of the PRX PLUS application, the facial skin was rinsed with water. Microneedling was subsequently performed over the full face using the PRX Pen (manufactured by Guangzhou Ekai Electronic Technology Co., Ltd., Guangzhou, China, for WiQo US; FDA cleared), with a fixed needle penetration depth of 0.25 mm. Immediately after microneedling, a topical formulation containing biosomes was evenly applied to the treated skin to support cutaneous uptake in the post-procedural window. Biosomes (AnteAGE^®^ Biosome Solution; Cellese Inc.) are non-cell-derived engineered synthetic lipid vesicles containing a recombinant protein cargo including KGF-1, KGF-2, TGFβ3, hBD3H, HGF, TRX, TIMP-1, NGF, SIRT-1, and SIRT-2, combined with a lipid blend of cholesterol, DOPC, and DOPS. Vesicles were produced by thin-film hydration followed by membrane extrusion, stabilized with trehalose, and lyophilized for storage at 4 °C; the product was reconstituted immediately before use. Particle concentration and size distribution were characterized by nanoparticle tracking analysis, lipid association by membrane-dye nano-flow cytometry, and morphology by cryo-transmission electron microscopy; detailed characterization data from a representative production lot are provided in Figure 1. For each treatment session, 5 mL of reconstituted product containing approximately 25 billion particles was applied.

No additional active agents were administered during the session. A standardized home-care protocol was prescribed throughout the study period and continued until the follow-up assessment. The regimen included WiQo Nourishing and Moisturizing Cream for Dry Skin (WiQo S.p.a.), applied twice daily, and WiQo Smoothing Fluid (5% glycolic acid) (WiQo S.p.a.), applied once daily in the evening prior to the moisturizer. The shea butter-based cream was intended to restore and maintain skin hydration and to counteract potential dehydration resulting from repeated microneedling sessions. The glycolic acid-containing fluid was used to promote gentle epidermal renewal and maintain adequate skin turnover while preserving skin barrier integrity. Overall, the home-care regimen was designed to reduce the risk of treatment-related adverse events, particularly skin dryness and dehydration, and to optimize the cutaneous environment for an effective regenerative response. Full INCI listings for both products are provided in Supplementary Table S1. No further in-clinic applications or adjunctive treatments were performed within the 24 h following each session.

### 2.4. Outcome Assessments

Clinical evaluations were conducted at two predefined time points: baseline (T0), immediately prior to the first treatment session, and post-treatment follow-up (T1), scheduled 15 days after the third session. This follow-up interval was selected to capture early epidermal and superficial dermal responses to the combined treatment protocol. Additionally, immediate post-procedural tolerability was assessed 5 min after the first treatment session.

Objective skin assessments were performed using the VISIA^®^ system (Canfield Scientific Inc., NJ, USA) and the Visioscope (Courage + Khazaka electronic GmbH, Cologne, Germany).

The VISIA^®^ system evaluates skin parameters using RBX^®^ (Red-Brown eXtraction) multispectral imaging technology, which separates the chromatic signatures of melanin and hemoglobin-related skin components under standardized illumination [10]. For each parameter, two metrics are reported: the feature count, representing the absolute number of pixels or discrete areas identified by the image analysis algorithm, and the score, a normalized index relative to an age- and phototype-matched reference population [11,12,13]. For all parameters assessed, a decrease in both feature count and score indicates improvement. The Visioscope quantifies surface skin characteristics under cross-polarized illumination; for all reported parameters, a decrease indicates improvement.

All image acquisitions were conducted in a controlled environment under standardized lighting conditions, with fixed camera positioning and consistent facial alignment in accordance with manufacturer recommendations. Measurements were obtained using the same devices and performed by the same operator to minimize inter-session variability. All treatments, eligibility assessments, Rubin photoaging grading, and instrumental measurements were performed by an experienced aesthetic medicine physician.

Immediate post-procedural tolerability was assessed using visual analogue scales (VAS) evaluating pain, discomfort, and burning sensation. In addition, redness-related parameters were measured immediately after treatment using the VISIA^®^ system.

Skin texture parameters were considered the primary outcomes of interest, based on their direct relationship with epidermal surface remodeling. All remaining VISIA and Visioscope parameters were treated as secondary and exploratory outcomes.

### 2.5. Statistical Analysis

Continuous variables were summarized using means and standard deviations, as well as medians and interquartile ranges (IQRs), while categorical variables were reported as absolute frequencies and percentages. Given the limited sample size, the ordinal nature of questionnaire-derived outcomes, and the anticipated non-normal distribution of several variables, a non-parametric analytical approach was adopted. Normality was formally checked using the Shapiro–Wilk test. Pre-post comparisons between baseline (T0) and post-treatment follow-up (T1) were performed using the Wilcoxon signed-rank test. Statistical significance was defined as a two-sided *p*-value < 0.05. No correction for multiple testing was applied, consistent with the exploratory nature of the study. As a sensitivity analysis, the Benjamini–Hochberg procedure was applied to control the false discovery rate (FDR) across all outcome parameters. No missing data were observed, and all enrolled participants completed both baseline and follow-up assessments. Statistical analyses were conducted using STATA version 19 (StataCorp., College Station, TX, USA).

## 3. Results

The study sample consisted of ten adult participants. The mean age was 46.7 years (SD 8.3), with a median age of 46.0 years [IQR 40.0–53.0]. According to the Rubin photoaging classification, participants were evenly distributed, with five subjects (50.0%) classified as Grade I and five (50.0%) as Grade II. Fitzpatrick skin phototypes ranged from II to VI. Phototypes II and III were the most represented (each accounting for 30.0% of the sample), followed by phototype IV (20.0%) and phototype VI (20.0%) (Table 1).

Immediate post-treatment evaluation showed low levels of acute procedural discomfort (Table 2). Subjective sensations of burning, pain, and discomfort were generally mild, with low absolute values across participants. Mean scores for burning sensation, discomfort, and pain were 0.7 (SD ±1.3), 1.2 (SD ±1.9), and 0.8 (SD ±1.0), respectively, with median values close to zero. Objective redness-related parameters exhibited moderate inter-individual variability, with mean redness feature and redness score values of 39.2 (SD ± 22.3) and 72.8 (SD ± 66.5), respectively.

Pre-post analyses showed changes across several objective skin-quality parameters (Table 3). Statistically significant differences were observed for texture-related parameters, pigmentation features, and redness metrics. Texture feature counts decreased from 2319.4 (SD ± 826.8) at baseline to 1855.7 (SD ± 796.9) at follow-up, corresponding to a 20.0% reduction (*p* = 0.009). The texture score decreased by 25.8%, from 19.4 (SD ±8.8) to 14.4 (SD ±7.9) (*p* = 0.005). Pigmented spot features decreased by 6.9% (*p* = 0.019), and the pigmentation score was reduced by 10.8% (*p* = 0.013). Redness-related parameters showed reductions in both feature counts and scores, with decreases of 42.3% (*p* = 0.012) and 33.3% (*p* = 0.022), respectively. Wrinkle feature counts decreased by 13.0% (*p* = 0.019), whereas the wrinkle score increased by 12.5%; this latter change did not reach statistical significance (*p* = 0.074). Visioscope analyses showed changes consistent with the VISIA-derived results. The number of spots decreased by 60.0% (*p* = 0.011), pores by 12.1% (*p* = 0.007), sebum levels by 35.7% (*p* = 0.011), and wrinkle severity by 9.2% (*p* = 0.009); all of these changes reached statistical significance.

The sensitivity analysis confirmed that all significant findings (*p* < 0.05) retained significance after correction (adjusted *p* range: 0.020–0.024).

Consistent with objective measurements, representative clinical and instrumental images demonstrated visible improvements in texture, redness, and pigmentation-related parameters (Figure 2).

## 4. Discussion

The present study evaluated the integration of Biosomes (engineered synthetic lipid vesicles) into a topical skin booster protocol. Overall, the findings indicate that this combined approach is well tolerated and associated with significant improvements in selected, objectively measurable skin-quality parameters, particularly skin texture, pigmentation-related features, and redness.

The most consistent and clinically relevant effects were observed in texture-related parameters, which showed significant reductions in both feature counts and global scores. Improvements in skin texture are of particular relevance in aesthetic practice, as they may reflect enhanced surface regularity and improved epidermal–dermal interaction rather than deep structural remodeling [2]. From a mechanistic perspective, these findings are compatible with the ability of PRX PLUS to enhance controlled epidermal activation while preserving barrier integrity [14]. Within the context of the combined protocol, the concurrent topical delivery of engineered synthetic lipid vesicles may provide additional biological signals relevant to tissue homeostasis and repair, although the independent contribution of each component cannot be determined from the present study.

Pigmentation-related outcomes demonstrated moderate but statistically significant improvements. Reductions in pigmented spot features and pigmentation scores suggest a modulatory effect on epidermal pigmentation dynamics. This interpretation is biologically plausible in the context of the combined protocol, given experimental evidence that EVs can influence melanocyte–keratinocyte crosstalk, skin turnover, and pigmentation pathways through paracrine signaling mechanisms [15,16]. Importantly, no clinically relevant pigmentary adverse events were observed, supporting the immediate tolerability of the combined protocol across different skin phototypes. No delayed or procedure-related adverse events were spontaneously reported.

Redness-related parameters exhibited some of the largest relative changes, particularly in feature-based measurements. Although the VISIA^®^ redness parameter captures the chromatic signature of red skin components and provides an indirect, instrument-based estimate of surface erythema, the observed reductions are consistent with an improvement in surface redness chromaticity, which may plausibly reflect a combined effect on vascular reactivity and cutaneous inflammation. The ammonium trichloroacetate-based skin booster is designed to promote controlled epidermal renewal without causing visible exfoliation or tissue damage [17], while EV-mediated signaling has been associated with anti-inflammatory and immunomodulatory effects in the skin [18,19]. Although vascular parameters can be influenced by transient procedural factors, the consistency of improvements across objective metrics is compatible with a reduction in inflammatory or vascular skin reactivity, although this interpretation should be verified in future studies with direct molecular or vascular measurements.

The concordance between VISIA^®^ and Visioscope findings strengthens the robustness of the results. Visioscope-derived reductions in spots, pore visibility, and sebum levels complement the VISIA data and support improvements in surface skin characteristics that are highly relevant in daily aesthetic practice. For wrinkle-related outcomes, a mixed pattern emerged: the VISIA^®^ wrinkles feature improved significantly, whereas the normalized wrinkles score did not, and the Visioscope wrinkle severity reduction, though significant, reflects a distinct optical dimension. This pattern is consistent with the superficial epidermal effect of the combined protocol, which may reduce the number of detectable wrinkle areas without proportionally altering their normalized severity relative to age-matched norms.

Immediate post-treatment assessments demonstrated low levels of pain, burning sensation, and discomfort, indicating favorable tolerability of the combined PRX PLUS and protocol incorporating engineered synthetic lipid vesicles. This aspect is clinically relevant, as procedural comfort and minimal downtime are key determinants of patient acceptance in minimally invasive aesthetic treatments. Previous reports suggest that EV-based formulations, when used as adjunctive agents, do not exacerbate inflammatory responses and may support post-procedural recovery [20,21], in line with the findings of the present study.

From a biological and clinical standpoint, the findings support a complementary interaction between the two treatment modalities. The combined protocol may act through complementary biological mechanisms: controlled epidermal activation induced by the skin booster component may enhance cutaneous receptivity, while the concurrent topical delivery of engineered synthetic lipid vesicles may provide additional modulatory signals relevant to intercellular communication, post-procedural inflammatory responses, and downstream skin quality outcomes [3,8]. This rationale aligns with the growing trend toward combination strategies in aesthetic medicine, in which skin booster treatments are paired with biologically active adjuncts to optimize clinical outcomes [22].

Our group has previously reported outcomes of the PRX PLUS and microneedling protocol without Biosomes in a comparable population [14]. Direct quantitative comparison between the two studies is not appropriate given differences in cohort selection and study design; nonetheless, this reference dataset underscores that the incremental contribution of Biosomes cannot be established from the current data alone. A prospective randomized study comparing the protocol with and without Biosomes, using identical instrumentation and standardized endpoints, represents the necessary next step.

Despite this promising evidence, this study has some limitations mainly due to its preliminary and exploratory nature. First, the small sample size combined with the assessment of multiple outcome parameters across two imaging systems substantially increases the risk of type I error and limits the generalizability of the findings and precludes definitive conclusions regarding efficacy. Although the post-hoc sensitivity analysis confirmed significant findings, the results should be interpreted as preliminary, requiring confirmation in adequately powered controlled studies. Second, the absence of a control group treated with PRX PLUS alone prevents isolation of the specific contribution of Biosomes to the observed effects. Third, the short follow-up period precludes assessment of the durability of the improvements. Fourth, no histological or molecular analyses were performed, limiting mechanistic insights into the observed clinical changes. Finally, as an observational study conducted under real-world conditions, potential confounding factors cannot be fully excluded. In particular, the standardized homecare regimen, which included a 5% glycolic acid fluid applied nightly throughout the study period, may independently have contributed to some improvements in skin quality and its specific contribution cannot be isolated. Future randomized controlled studies with larger sample sizes and longer follow-up are warranted to confirm these findings and better define the role of engineered synthetic lipid vesicles within combination aesthetic protocols.

In conclusion, the integration of engineered synthetic lipid vesicles into a topical skin booster protocol was associated with favorable immediate tolerability and significant improvements in selected objective skin-quality parameters, particularly texture, pigmentation-related features, and redness. Although preliminary and conducted without a comparator arm, these findings support the rationale for further investigation of engineered synthetic lipid vesicles as active adjuncts in minimally invasive aesthetic strategies focused on skin-quality enhancement. The observed improvements in procedural tolerability highlight a potentially important role for these vesicles in optimizing patient experience in minimally invasive aesthetic treatments.

## Figures and Tables

**Figure 1:**
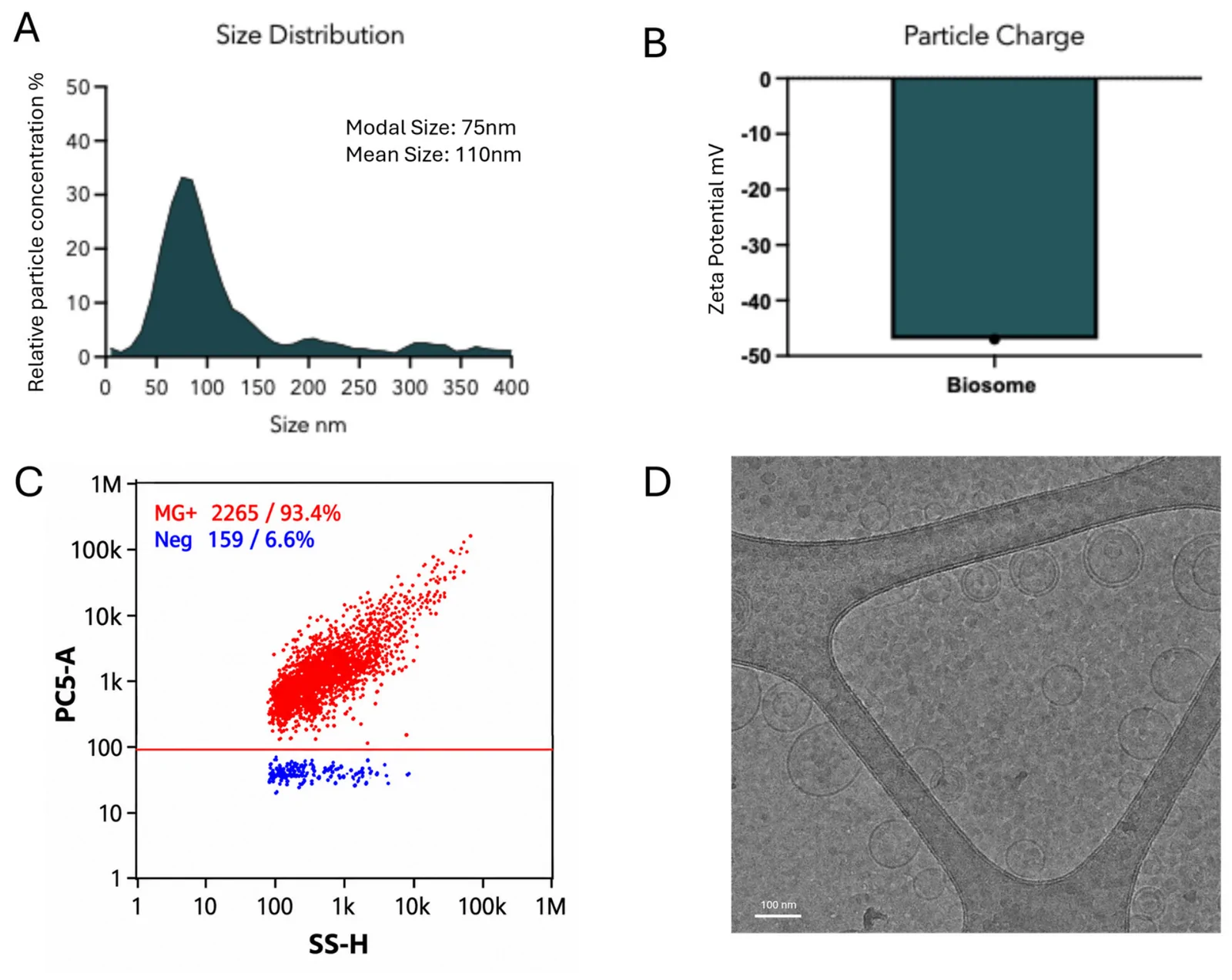
Physicochemical and structural characterization of engineered synthetic lipid vesicles (Biosomes). (**A**) Representative particle-size distribution of reconstituted Biosomes determined by nanoparticle tracking analysis and expressed as relative particle concentration across the measured size range. The modal and mean particle diameters are indicated in the panel. (**B**) Zeta-potential measurement demonstrating a negative particle surface charge. (**C**) Nano-flow cytometric analysis following staining with the lipid-membrane dye MemGlow™ 488 (Cytoskeleton Inc., Denver, CO, USA). The horizontal line indicates the fluorescence-positivity threshold. MemGlow-positive events are shown in red (2265 events; 93.4%), and negative events are shown in blue (159 events; 6.6%). (**D**) Representative cryo-transmission electron microscopy image showing approximately spherical, membrane-delimited vesicles. Scale bar, 100 nm. Data shown are from a representative Biosome production lot manufactured using the standardized production process and used in the clinical study.

**Figure 2:**
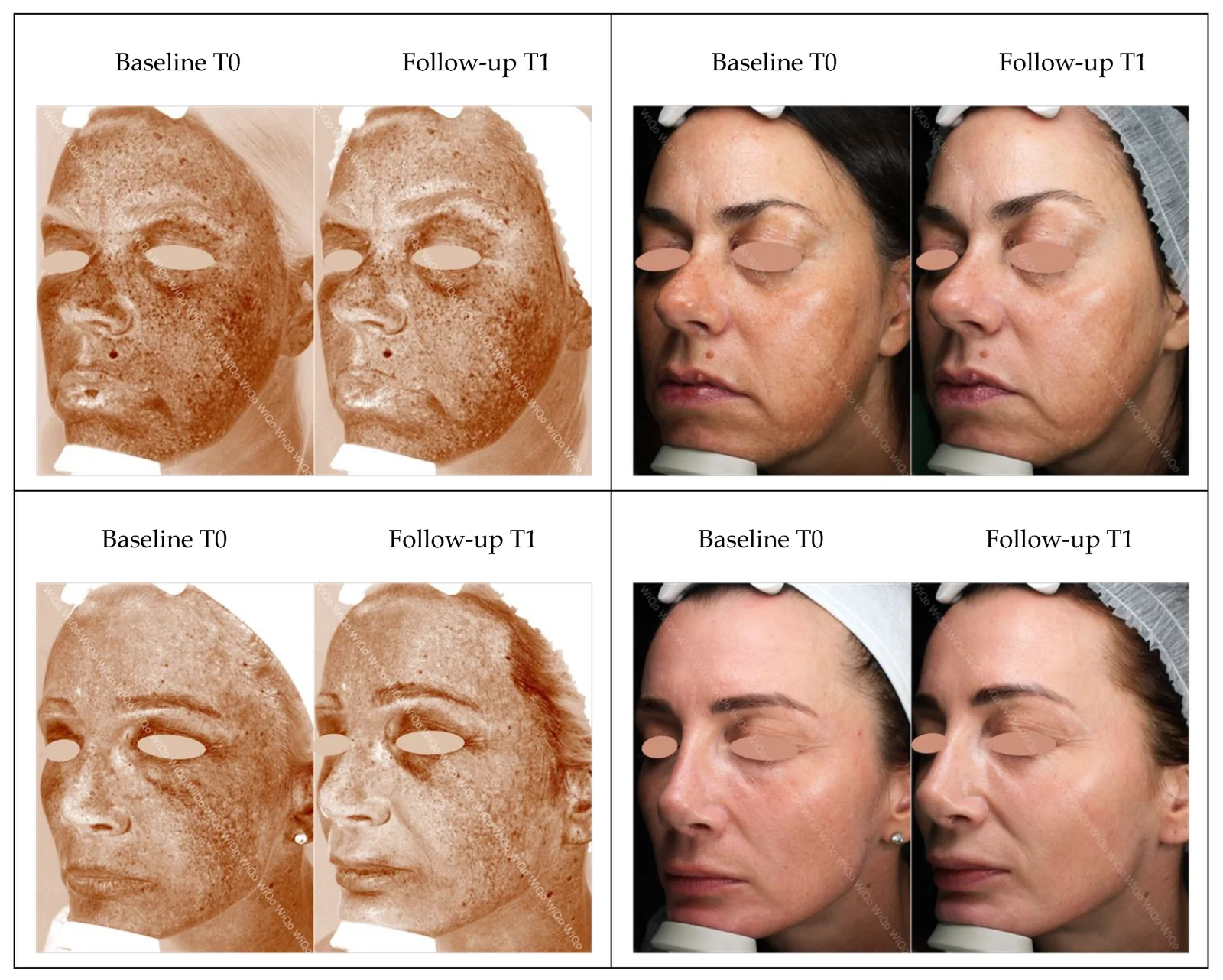
Standardized facial images and corresponding VISIA^®^/instrumental pigmentation images obtained before (**left side**) and after (**right side**) the treatment protocol. Images show visible improvements in overall skin quality, including more even tone, reduced apparent redness, improved surface texture, and reduced pigmentation contrast.

**Table 1:** Patients’ characteristics.

Total Sample	10
Age (years), mean (SD) [median, 25th–75th]	46.7 (8.3) 46.0 [40.0, 53.0]
Rubin Grade, *n* (%)	
I	5 (50.0)
II	5 (50.0)
Fitzpatrick phototype, n (%)	
Type I—Very fair skin; always burns, never tans	0 (0.0)
Type II—Fair skin; usually burns, tans minimally	3 (30.0)
Type III—Medium/light brown skin; sometimes mild burn, gradually tans	3 (30.0)
Type IV—Olive/moderate brown skin; rarely burns, tans well	2 (20.0)
Type V—Dark brown skin; very rarely burns, tans very easily	0 (0.0)
Type VI—Deeply pigmented dark brown to black skin; never burns	2 (20.0)

Results are reported as frequencies and percentages, unless otherwise specified. SD: Standard Deviation.

**Table 2:** Immediate Post-evaluation.

	Total Sample (n = 10)
Burning sensation (VAS 0–10)	0.7 (1.3); 0.0 [0.0, 1.0]
Discomfort (VAS 0–10)	1.2 (1.9); 0.0 [0.0, 2.0]
Pain (VAS 0–10)	0.8 (1.0); 0.5 [0.0, 1.0]
Redness features *	39.2 (22.3); 38.1 [25.5, 59.4]
Redness score *	72.8 (66.5); 61.0 [16.0, 113.0]

Results are reported as mean (SD); median [IQR]-* Measured through VISIA^®^.

**Table 3:** Pre-post comparison measured with VISIA^®^ and Visioscope.

Parameter		T0 (*n* = 10)	T1 (*n* = 10)	% Change	*p*-Value	MD (95%CI)	r
*VISIA^®^ outcomes*							
BS	Feature	180.5 (75.7) [181.5; 97–241]	178.3 (78.3) [162; 111–258]	−1.2%	0.878	−2.20 [−29.71; +25.31]	0.048
	Score	23.0 (8.7) [22.9; 15.1–27.5]	22.0 (7.9) [19.4; 16–22.7]	−4.3%	0.386	−0.95 [−3.63; +1.73]	0.274
UV	Feature	289.0 (59.5) [315.5; 243–325]	287.8 (76.6) [313; 258–332]	−0.4%	0.959	−1.20 [−34.51; +32.11]	0.016
	Score	21.4 (8.4) [20.9; 13.3–26.8]	22.0 (8.3) [22.7; 17.6–29.7]	+2.8%	0.799	+0.61 [−2.37; +3.60]	0.081
Wrinkle	Feature	100.0 (37.1) [96; 92–122]	87.0 (25.4) [91.5; 76–101]	−13.0%	0.019 (p_BH_ = 0.023)	−13.00 [−23.70; −2.30]	0.743
	Score	35.1 (10.0) [36.8; 27.1–41.3]	39.5 (9.1) [39.6; 37.3–47.1]	+12.5%	0.074 (p_BH_ = 0.074)	+4.43 [−0.63; +9.49]	0.564
Texture	Feature	2319.4 (826.8) [2438.5; 1891–2919]	1855.7 (796.9) [1619; 1293–2755]	−20.0%	0.009 (p_BH_ = 0.020)	−463.70 [−694.88; −232.52]	0.822
	Score	19.4 (8.8) [19.2; 14.4–26.2]	14.4 (7.9) [10.8; 8.5–21.8]	−25.8%	0.005 (p_BH_ = 0.020)	−5.02 [−7.31; −2.74]	0.886
Pores	Feature	492.0 (152.4) [542.5; 351–578]	484.8 (190.5) [458; 351–658]	−1.5%	0.445	−7.20 [−105.38; +90.98]	0.242
	Score	13.6 (4.4) [14.6; 9.6–16.8]	13.1 (5.6) [11.9; 8.4–18.7]	−3.7%	0.445	−0.52 [−3.73; +2.68]	0.242
Spots	Feature	150.2 (32.4) [158.5; 120–177]	139.9 (27.9) [139; 118–169]	−6.9%	0.019 (p_BH_ = 0.023)	−10.30 [−18.68; −1.92]	0.742
	Score	35.3 (7.6) [36.4; 29.3–43.1]	31.5 (7.1) [31.7; 26.3–38.1]	−10.8%	0.013 (p_BH_ = 0.020)	−3.77 [−6.24; −1.30]	0.790
Redness	Feature	56.5 (35.0) [58.5; 30–71]	32.6 (21.3) [33; 15–42]	−42.3%	0.012 (p_BH_ = 0.020)	−23.90 [−38.13; −9.67]	0.790
	Score	18.9 (10.9) [17; 12.9–19.7]	12.6 (6.9) [11.4; 10.3–14.5]	−33.3%	0.022 (p_BH_ = 0.024)	−6.29 [−11.32; −1.26]	0.725
*Visioscope outcomes*							
Spots		(0.7) [1; 0.2–1.5]	0.4 (0.5) [0.3; 0–0.5]	−60.0%	0.011 (p_BH_ = 0.020)	−0.60 [−1.04; −0.16]	0.804
Pores		3.3 (1.0) [3.4; 2.4–4.4]	2.9 (1.2) [3; 1.9–4]	−12.1%	0.007 (p_BH_ = 0.020)	−0.45 [−1.22; +0.32]	0.846
Sebum		1.4 (2.0) [0.4; 0.2–2.3]	0.9 (1.8) [0.3; 0.1–0.6]	−35.7%	0.011 (p_BH_ = 0.020)	−0.50 [−1.09; +0.09]	0.805
Wrinkles		7.6 (2.5) [8.5; 6.2–9.5]	6.9 (2.5) [7; 5.6–9.7]	−9.2%	0.009	−0.65 [−1.11; −0.19]	0.822

Results are reported as mean (SD) [median, 25th–75th]. p_BH_: FDR. Mean difference (MD) and 95% CI computed from paired differences (T1 − T0). Effect size $\left|r\right|\;=\left|\;Z/\sqrt{n}\right|$ from Wilcoxon signed-rank Z statistic (0.1 = small, 0.3 = medium, 0.5 = large).

## Data Availability

Data are available upon request from the corresponding author, provided that their use complies with applicable Italian and European privacy legislation.

## Supplementary Materials

The following supporting information can be downloaded at: https://www.mdpi.com/article/10.3390/biomedicines14102172/s1, Table S1. Home-care regimen products ingredients list.

## Abbreviations

The following abbreviations are used in this manuscript:

BS	Brown spots
EV	Extracellular vesicle
IQR	Interquartile range
SD	Standard Deviation
sEV	Small extracellular vesicle
STROBE	Strengthening the Reporting of Observational Studies in Epidemiology
UV	Ultraviolet
VAS	Visual analogue scale
