# Supplementary material for: Safety and Preliminary Clinical Outcomes of Integrating Engineered Synthetic Lipid Vesicles (Biosomes) in a Skin Booster Protocol: A Prospective Observational Study
Source: Biomedicines. 2026 Sep 25;14(10):2172. doi: 10.3390/biomedicines14102172 (PMC13701172; doi:10.3390/biomedicines14102172)
Supplement: Supplementary file 1 [file biomedicines-14-02172-s001.zip › biomedicines-4558196-supplementary.pdf]

Supplementary materials

Table S1. Home-care regimen products ingredients list

**WIQO NOURISHING AND MOSITURIZING CREAM FOR DRY SKIN**: AQUA (WATER), BUTYROSPERMUM PARKII (SHEA) BUTTER, PROPYLENE GLYCOL, PARAFFINUM LIQUIDUM (MINERAL OIL), GLYCERYL STEARATE SE, HYDROGENATED POLYDECENE, CETYL ALCOHOL, BIS-PEG-18 METHYL ETHER DIMETHYL SILANE, SIMMONDSIA CHINENSIS (JOJOBA) SEED OIL, TRITICUM VULGARE (WHEAT) GERM OIL, VITIS VINIFERA (GRAPE) SEED OIL, ECHINACEA ANGUSTIFOLIA (ECHINACEA) ROOT EXTRACT, BUTYL METHOXYDIBENZOYLMETHANE, OCTOCRYLENE, BENZYL ALCOHOL, PENTAERYTHRITYL TETRA-DI-T-BUTYL HYDROXYHYDROCINNAMATE, TOCOPHERYL ACETATE, RETINYL PALMITATE, HELIANTHUS ANNUUS (SUNFLOWER) SEED OIL, DEHYDROACETIC ACID, SODIUM GLUCONATE, PARFUM (FRAGRANCE), BENZYL SALICYLATE, LINALOOL, LIMONENE, GERANIOL, CITRAL, CITRONELLOL, HYDROXYCITRONELLAL, CINNAMYL ALCOHOL, HEXYL CINNAMAL, AMYL CINNAMAL, ISOEUGENOL, LINALYL ACETATE, AMYL SALICYLATE, CITRUS LIMON PEEL OIL, TERPINEOL, PINENE.
**WIQO SMOOTHING FLUID** WATER (AQUA), DIMETHICONE, GLYCOLIC ACID, STEARETH-2, PEG-8 DISTEARATE, STEARETH-21, GLYCERIN, TOCOPHERYL ACETATE, DIMETHICONOL, BENZYL ALCOHOL, CETEARYL ALCOHOL, SODIUM HYDROXIDE, DEHYDROACETIC ACID, PHYTIC ACID, LECITHIN, TOCOPHEROL, ASCORBYL PALMITATE, CITRIC ACID.
